# Accelerometry-based comparison of functional outcomes across total and partial knee and hip arthroplasty: a propensity-matched UK Biobank Study

**DOI:** 10.1302/2046-3758.1510.BJR-2025-0603.R1

**Published:** 2026-10-13

**Authors:** Sevasti Panagiota Glynou, Omar Musbahi, Sara Sousi, Justin P. Cobb

**Affiliations:** 1 MSk Lab, Imperial College London, London, UK; 2 Institute of Global Health Innovation, Imperial College London, London, UK

**Keywords:** Hip arthroplasty, Knee arthroplasty, Physical activity, Accelerometry, Gait analysis, hip and knee arthroplasties, accelerometry, knee, unicompartmental knee arthroplasty (UKA), total knee arthroplasty (TKA), cadence, hips, hip and knee arthroplasties, propensity score matching, total hip arthroplasty (THA)

## Abstract

**Aims:**

To evaluate objectively measured physical activity after hip and knee arthroplasty using accelerometry, by comparing bone-preserving procedures with total arthroplasties and benchmarking both against non-arthritic controls.

**Methods:**

This observational study was conducted within the UK Biobank. Adults aged 40 to 69 years with total knee arthroplasty (TKA), unicompartmental knee arthroplasty (UKA), total hip arthroplasty (THA), or hip resurfacing (HR) were identified through linked hospital records and completed seven-day wrist-worn accelerometry (Axivity AX3). Outcomes included adjusted daily step count (primary), peak cadence, moderate-to-vigorous physical activity (MVPA), and Euclidean Norm Minus One (ENMO). Surgical groups were propensity score-matched to non-arthritic controls on demographics, lifestyle factors, and comorbidities. Direct comparisons between procedure types were examined using both propensity-matched analyses and complementary multivariable regression, and adjusted for demographic and clinical covariates. Subgroup analyses considered < 12 months and ≥ 12 months post-surgery.

**Results:**

A total of 2,283 participants were included (TKA = 860, UKA = 162, THA = 1,168, HR = 93). Compared with matched controls, TKA and THA patients showed persistently lower activity, while UKA and HR achieved levels similar to controls. In the knee cohort, UKA patients walked significantly more steps/day than TKA (9,150 vs 7,920; + 15.5%, p < 0.001) with higher cadence. In the hip cohort, HR patients outperformed THA (10,122 vs 9,139 steps/day; + 10.6%, p = 0.032) and demonstrated higher peak cadence, and HR remained comparable to controls. All groups fell within the ‘somewhat active’ to ‘active’ step-count range (7,500 to 12,499 steps/day).

**Conclusion:**

UKA and HR are associated with higher postoperative activity compared with total arthroplasty (TKA, THA). Accelerometry provides an objective, scalable assessment of functional recovery, highlighting procedure-specific differences that should be considered alongside implant longevity when selecting surgical options.

Cite this article: *Bone Joint Res* 2026;15(10):1213–1227.

## Article focus

- To compare objectively measured physical activity after hip and knee arthroplasty using accelerometry.
- To evaluate differences between total arthroplasty (THA, TKA) and bone-preserving procedures (hip resurfacing (HR), unicompartmental knee arthroplasty (UKA).
- To benchmark activity outcomes against propensity-matched non-arthritic controls in UK Biobank.

## Key messages

- Patients undergoing HR and UKA achieved activity levels comparable to healthy controls, whereas THA and TKA patients remained less active.
- UKA patients walked 1,230 more steps/day compared to TKA (9,150 vs 7,920; + 15.5%), while HR patients walked 973 more steps/day compared to THA (10,112 vs 9,139; + 10.6%), with higher peak cadence observed in both comparisons.
- Accelerometry provides scalable, objective functional assessment, revealing procedure-specific differences which are not captured by patient-reported outcome measures.

## Strengths and limitations

- This study used a large, population-based sample (n = 2,283) with device-measured activity and robust propensity score matching.
- This is the first large-scale, propensity score-matched comparison of bone-preserving versus total arthroplasty using accelerometry.
- Cross-sectional accelerometer data (7 days) limits assessment of individual recovery trajectories.

## Introduction

Osteoarthritis (OA) is a leading cause of disability globally. In the UK alone, approximately 5.4 million people are affected by knee OA and 3.2 million by hip OA.^1,2^ As the ageing population and obesity rates increase, the demand for joint arthroplasty continues to rise.^3^ By 2035, hip and knee arthroplasties in the UK are projected to increase by over 40%, placing considerable strain on surgical services and rehabilitation resources across the country.^4^

Total knee arthroplasty (TKA), unicompartmental knee arthroplasty (UKA), total hip arthroplasty (THA), and hip resurfacing (HR) are well-established surgical interventions for end-stage OA. While all aim to improve function, they differ in their biomechanical design, tissue preservation, and postoperative recovery trajectories, which may explain the substantial difference in long-term mortality seen following the different procedures.^5^ Although several comparative studies have examined these procedures in controlled settings, large-scale real-world comparisons using objective measures remain limited.^6,7^

Historically, assessment of postoperative outcomes have relied heavily on patient-reported outcome measures (PROMs). While these are clinically validated for evaluating perceived postoperative pain, satisfaction, and function, they are limited by recall bias, ceiling effects, and inter-patient variability.^8,9^ In response, over the last decade, there is growing interest in integrating objective gait metrics to enhance functional assessment. Laboratory-based gait analysis systems, such as force platforms and marker-based systems, provide precise biomechanical data, but are resource-intensive and limited to short observation periods. Wearable technologies now allow scalable, continuous monitoring in real-world settings. Devices such as wrist- or hip-worn accelerometers can track gait parameters including step count, gait speed, cadence, range of motion (ROM), and kinetic variability over extended periods.^10,11^

Several gait analysis studies suggest that joint-preserving procedures, including UKA and HR, are associated with more favourable biomechanical outcomes than their total arthroplasty counterparts. Nonetheless, the evidence across the literature remains heterogeneous. Gerhardt et al^6^ reported that HR achieved higher top walking speeds (2.03 m/s vs 1.92 m/s), more symmetrical weight distribution (adjusted difference: 144 N; 95% CI 20 to 261), and greater hip flexion (adjusted difference: 8°; 95% CI 1.7 to 14) than those with THA. However, in a ten-year follow-up of a 32-patient study, Jelsma et al^12^ reported that differences in physical activity (PA) measured between HR and THA patients, such as daily step count (HR: 5,546 vs THA: 4,583; p = 0.6), sit–stand transfers (39 vs 37; p = 0.7), and cadence (102 vs 98 steps/min; p = 0.3) failed to reach statistical significance.

Similarly, Wiik et al^7^ showed that UKA achieved a 13% increase in walking speed (7.0 km/h vs 6.2 km/h, p = 0.001), with longer strides and shorter stance times than those undergoing TKA. A recent systematic review by Zhang et al^13^ supported these findings, noting consistent advantages for UKA in stride length, symmetry, and ROM across most studies, despite similar PROM scores. In contrast, a meta-analysis of seven non-randomized studies found no differences in gait parameters, other than a modest stride length advantage for UKA (mean difference 0.41 cm, p = 0.04).^14^

A recent study by Small et al,^15^ using UK Biobank data, showed that individuals who underwent hip or knee arthroplasty were more active than those with untreated end-stage OA, however their activity levels remained lower than those of non-arthritic controls after multivariable adjustment for confounders. Notably, their analysis did not differentiate between specific types of knee and hip procedures, limiting the ability to draw conclusions by specific surgical type. Although these findings are encouraging, most comparative studies to date have been constrained by small sample sizes, limited follow-up durations, and reliance on self-reported outcomes. The UK Biobank, with its integration of clinical, surgical, and device-based PA data offers an opportunity to evaluate postoperative function across procedures using large-scale, objective data. This study uses accelerometer-derived gait data from the UK Biobank to compare postoperative PA between UKA and TKA, and HR and THA, benchmarked against propensity-matched non-arthritic controls.

## Methods

### Data source and study population

The data used in this study are available via application to the UK Biobank under project ID 194406.^16^ The data are not publicly available but can be accessed by approved researchers conducting health-related research in the public interest. The UK Biobank is a large-scale, community-based dataset containing medical, surgical, and activity-monitoring data from over 500,000 patients aged 40 to 69 years at recruitment, sourced from the UK’s NHS.^17,18^ Participants wore an Axivity AX3 triaxial accelerometer (Axivity, UK) on their dominant wrist over a continuous seven-day period, with data sampled at 100 Hz and a dynamic range of ± 8 g. Acceleration signals were calibrated to local gravity, ensuring consistency across devices and conditions. Data processing procetime from operation to dures included resampling and low-pass filtering to remove sensor noise and gravitational components. Non-wear periods were removed to avoid skewing activity data, while imputation methods were employed to fill any detected gaps and preserve the time-of-day integrity of activity patterns.^18,19^

For inclusion in the dataset, participants were required to wear the accelerometer for a minimum of 72 hours, or at least one hour of wear in each hour of the day over the seven-day period.^18^

The UK Biobank sources phenotypic, demographic, diagnostic, and operational data from NHS Hospital Episode Statistics (HES), Patient Episode Database for Wales (PEDW) Admitted Patient Care dataset for Wales, and Scotland’s Admitted Patient Care dataset. Inpatient records date back to 1997 for England, 1998 for Wales, and 1981 for Scotland, offering extensive longitudinal coverage across the UK.^20^

### Accelerometer data analysis

Accelerometer data was processed using the OxWearables stepcount package (v. 3.11.0) to extract core PA metrics, including step count, walking duration, and cadence (steps per minute). PA measures were derived using established UK Biobank accelerometer processing methods that have been previously described and validated.^18,21^ Missing data points were imputed by averaging the same timepoint across available days to account for variation in device wear time. This approach was used to derive adjusted daily step count and peak cadence values, representing time-of-day-normalized estimates of activity rather than raw observed values. Non-adjusted values reflect raw observed activity, whereas adjusted values account for incomplete wear and provide a more representative estimate of habitual activity. The stepcount package uses a self-supervised machine learning algorithm tailored for wrist-worn accelerometer data, trained and fine-tuned on data from the Axivity AX3 device used by the UK Biobank. Output variables included minutely, hourly, and daily summaries of both adjusted and non-adjusted step counts. The adjusted daily step count, adjusted peak cadence, and Euclidean Norm Minus One (ENMO) acceleration were used in this study.

Additional activity data was classified into categories of light PA, and moderate-to-vigorous physical activity (MVPA) using a machine-learning model incorporating random forest and hidden Markov algorithms.^22^ This model was developed by Walmsley et al^23^ and provides enhanced classification accuracy for the time spent in each activity level.

### Data acquisition, preparation, and preprocessing

Data were accessed via the UK Biobank Research Analysis Platform (DNAnexus), including phenotypic, demographic, HES, diagnostic, procedural, and accelerometer datasets.

Phenotypic and demographic included age, sex, ethnicity (white vs non-white), BMI, smoking (never, former, current), alcohol intake (none, < 3 units/day, ≥ 3 units/day), education (school leaver, further, higher), and deprivation index (quintiles). Comorbidity was calculated using the Charlson Comorbidity Index (CCI) based on inpatient diagnoses within a five-year lookback period from the date of accelerometer wear.^24,25^

The diagnostic dataset included International Classification of Diseases Tenth Revision (ICD-10) codes and diagnosis dates, enabling identification of participants with knee and hip OA. For temporal alignment with accelerometer data, only OA diagnoses that preceded the accelerometer wear period were included.^15,25^ See Supplementary Table 1 for ICD-10 codes.

Surgical procedures were identified using Office of Population Censuses and Surveys Classification of Interventions and Procedures (OPCS-4) codes, including procedure type, date, and admission/discharge records.^26,27^ Primary TKA, UKA, THA, and HR were included. Exclusion criteria were revision surgery prior to accelerometer wear, combined hip and knee arthroplasties, mixed procedure types (e.g. UKA and TKA), or more than two primary operations at the same joint and laterality. To confirm OA as the surgical indication, a recorded OA diagnosis had to precede the operation. Participants with documented diagnoses of both hip and knee OA prior to their first arthroplasty were excluded to allow joint-specific analysis. OPCS-4 procedure codes used to define surgical cohorts are provided in Supplementary Tables 2 to 5.

### Ethical approval and informed consent

All analyses were conducted in accordance with the ethical standards and regulatory requirements of the UK Biobank protocol and broader research governance guidelines. The UK Biobank study was approved by the North West multicentre Research Ethics Committee (REC reference: 11/NW/0382). Informed consent was obtained from all participants at the time of recruitment into the UK Biobank.

### Cohort generation and matching

The accelerometer, diagnostic, operational, and phenotypic datasets were merged. To ensure high data quality, patients were filtered based on accelerometer wear quality (minimum three days of seven-day wear with good calibration). To ensure data completeness and allow for complete case analysis, participants with incomplete demographic data, including “Prefer not to answer” or “None of the above” responses were excluded. Upon data cleaning, participants were classified into two groups: non-arthritic with no operation, and arthritic with operation.

To minimize confounding effects when comparing outcomes across groups, propensity score matching (PSM) was conducted using the MatchIt package (v. 4.5.5) in R (R Foundation for Statistical Computing, Austria).^28^ All surgical participants were separately matched to participants who neither had OA nor underwent surgery. Propensity scores were estimated using logistic regression with a logit link function. Nearest neighbour matching was applied based on the logit of the propensity score without arthroplasty, selecting individuals with the closest propensity scores.

Separate matching strategies were used depending on the comparison. For comparisons between arthroplasty groups and non-arthritic controls, 1:1 matching was applied. Matching covariates included age, sex, BMI, season of accelerometer wear, ethnicity, socioeconomic status, education, alcohol consumption, smoking status, and Charlson Comorbidity Index (CCI) score.^15,24^ For direct comparisons between arthroplasty subtypes (TKA vs UKA and THA vs HR), a 4:1 matching ratio was used, whereby each UKA or HR participant was matched to up to four TKA or THA participants, respectively, to maximize retention of the smaller groups while maintaining covariate balance. Matching covariates in this analysis included age, sex, BMI, season of accelerometer wear, CCI score, time from surgery to accelerometer assessment, and alcohol consumption.

### Workflow execution and software use

The analytical workflow began with data access and retrieval from the UK Biobank datasets using terminal-based commands on the DNAnexus cloud, supported by the DNAnexus Platform SDK (dx-toolkit). Data preprocessing, visualization, and statistical analyses were completed using R (v. 4.4.1), and Python (v. 3.10.9) was required for the stepcount package. Full diagnostic, procedural, and comparative analyses are provided in Supplementary Tables 1 to 7.

### Statistical analysis

The TKA, UKA, THA, and HR participants’ values were compared with the propensity matched health (non-arthritic) participants, with regard to their demographics and the PA measures, namely, light PA, MVPA, adjusted daily step count, adjusted peak cadence, and ENMO. Subsequently, the knee and hip cohorts were compared with each other, initially unmatched. Unmatched comparisons of accelerometer-derived PA outcomes are presented in Supplementary Table 7. Subsequently, their outcomes were compared using PSM on age, sex, BMI, CCI Score, and time from operation to accelerometer worn with a 4:1 ratio. Subsequently, they were also compared to the healthy non-arthritic cohort. Categorical variables were compared using Pearson’s chi-squared test, while continuous variables and accelerometer-derived physical activity outcomes were compared using the Kruskal-Wallis rank sum test.

Subgroup analyses were conducted based on time from surgery to accelerometer assessment (< 12 months and ≥ 12 months) to explore differences across postoperative phases. This threshold was selected to distinguish an early postoperative recovery period from a later, more functionally stable phase, in line with prior evidence suggesting that gait characteristics continue to evolve during the first postoperative year.^29,30^ These analyses were exploratory and not intended to imply uniform recovery trajectories.

In addition to PSM, complementary multivariable regression analyses were performed for the direct comparisons between UKA and TKA, and between HR and THA, using the full eligible unmatched cohort. These analyses were undertaken to retain the full sample, reduce loss of information caused by matching, and assess whether the observed associations remained consistent after adjustment for measured confounders.

## Results

A flowchart outlining the derivation of the final study cohort, including inclusion and exclusion steps, is shown in Figure 1. A total of 2,283 participants were included: 860 TKA, 162 UKA, 1,168 THA, and 93 HR procedures.

**Fig. 1:**
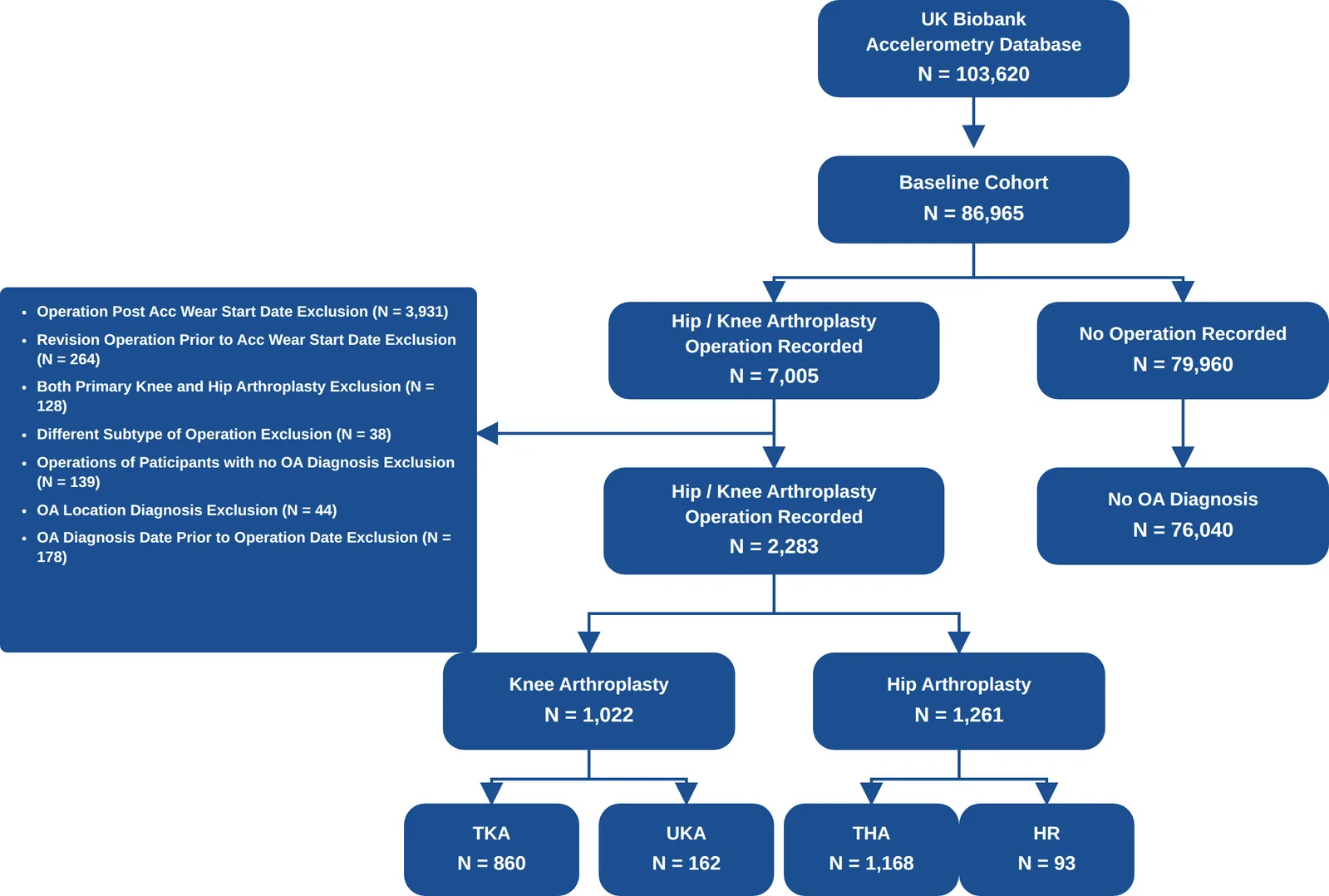
Flow diagram illustrating the selection process for the final study cohort from the UK Biobank accelerometry (acc) dataset, including inclusion and exclusion criteria for participants with hip or knee arthroplasty. HR, hip resurfacing; OA, osteoarthritis; THA, total hip arthroplasty; TKA, total knee arthroplasty; UKA, unicompartmental knee arthroplasty.

### Demographic characteristics of knee and hip surgery cohorts compared to healthy controls

After 1:1 PSM to non-osteoarthritic controls, demographic and clinical characteristics were broadly well-balanced across all cohorts (Table I). In the knee cohort, TKA patients had slightly higher BMI than their matched non-OA controls (30.0 vs 29.2 kg/m², p = 0.007; SMD 0.065), while no significant demographic differences were observed between UKA and controls (Table I). In the hip cohort, THA participants were broadly comparable to matched controls across baseline variables, although CCI differed statistically (p < 0.001; SMD 0.098). HR participants were also similar to matched controls, with some residual imbalance reflected in SMD values (Table I). Pre-matched and post-matched comparative analyses between arthroplasty types are presented in Supplementary Table 6. Baseline characteristics of the 4:1 propensity matched knee and hip arthroplasty cohorts are shown in (Table II).

**Table I.** Demographic and clinical characteristics of knee and hip arthroplasty cohorts after 1:1 propensity score matching to non-osteoarthritic controls.

Characteristic	Knee cohort								Hip cohort							
	TKA cohort				UKA cohort				THA cohort				HR cohort			
	Non OA (n = 860)	TKA (n = 860)	p-value	SMD	Non OA (n = 162)	UKA (n = 162)	p-value	SMD	Non OA (n = 1,168)	THA (n = 1,168)	p-value	SMD	Non OA (n = 93)	HR (n = 93)	p-value	SMD
**Sex, n (%)**			0.699†	0.019			0.738†	0.037			0.213†	0.052			0.645†	0.068
Female	453 (52.7)	461 (53.6)			90 (55.6)	87 (53.7)			724 (62.0)	753 (64.5)			34 (36.6)	31 (33.3)		
Male	407 (47.3)	399 (46.4)			72 (44.4)	75 (46.3)			444 (38.0)	415 (35.5)			59 (63.4)	62 (66.7)		
Median age, yrs (IQR)*	69.0 (66.0 to 72.0)	68.5 (65.0 to 72.0)	0.111‡	0.072	67 (62 to 71)	67 (62 to 71)	0.974‡	0.017	68.0 (64.0 to 71.0)	68.0 (64.0 to 71.0)	0.304‡	0.036	64 (57 to 68)	64 (59 to 67)	0.717‡	0.082
**Median BMI, kg/m^2^ (IQR)**	29.2 (26.0 to 33.6)	30.0 (27.0 to 33.7)	0.007‡	0.065	28.6 (25.3 to 32.1)	28.7 (26.4 to 32.9)	0.467‡	0.029	26.6 (24.1 to 29.5)	26.7 (24.2 to 29.9)	0.304‡	0.025	26.8 (24.1 to 29.2)	27.3 (24.7 to 29.8)	0.521‡	0.040
**Ethnicity, n (%)**			0.587†	0.100			> 0.999†	0.010			> 0.999†	0.056			> 0.999†	0.085
White	846 (98.4)	843 (98.0)			158 (97.5)	158 (97.5)			1,159 (99.2)	1,159 (99.2)			92 (98.9)	91 (97.8)		
Asian or Asian British	3 (0.3)	8 (0.9)			0 (0.0)	3 (1.9)			2 (0.2)	2 (0.2)			0 (0)	0 (0)		
Black or Black British	7 (0.8)	3 (0.3)			2 (1.2)	0 (0.0)			3 (0.3)	4 (0.3)			1 (1.1)	1 (1.1)		
Chinese	0 (0)	0 (0)			0 (0.0)	0 (0.0)			1 (0.1)	0 (0.0)			0 (0)	0 (0)		
Mixed	2 (0.2)	3 (0.3)			1 (0.6)	1 (0.6)			2 (0.2)	1 (0.1)			0 (0)	1 (1.1)		
Other ethnic group	2 (0.2)	3 (0.3)			1 (0.6)	0 (0.0)			1 (0.1)	2 (0.2)			0 (0)	0 (0)		
**Median CCI score (IQR)**	0.00 (0.00 to 0.00)	0.00 (0.00 to 0.00)	0.099‡	0.028	0.00 (0.00 to 0.00)	0.00 (0.00 to 1.00)	0.466‡	0.061	0.00 (0.00 to 0.00)	0.00 (0.00 to 0.00)	< 0.001‡	0.098	0.00 (0.00 to 0.00)	0.00 (0.00 to 0.00)	0.579‡	0.153
**Alcohol consumption, n (%)**			0.213†	0.085			0.514†	0.128			0.278†	0.066			0.638†	0.133
Never	78 (9.1)	59 (6.9)			8 (4.9)	6 (3.7)			71 (6.1)	62 (5.3)			1 (1.1)	2 (2.2)		
< 3 d/w	383 (44.5)	402 (46.7)			75 (46.3)	67 (41.4)			456 (39.0)	492 (42.1)			25 (26.9)	29 (31.2)		
≥ 3 d/w	399 (46.4)	399 (46.4)			79 (48.8)	89 (54.9)			641 (54.9)	614 (52.6)			67 (72.0)	62 (66.7)		
**Smoking, n (%)**			0.243†	0.081			0.814†	0.071			0.961†	0.012			0.961†	0.045
Never	416 (48.4)	414 (48.1)			58 (35.8)	63 (38.9)			639 (54.7)	637 (54.5)			56 (60.2)	58 (62.4)		
Previous	383 (44.5)	401 (46.6)			90 (55.6)	87 (53.7)			471 (40.3)	470 (40.2)			34 (36.6)	32 (34.4)		
Current	61 (7.1)	45 (5.2)			14 (8.6)	12 (7.4)			58 (5.0)	61 (5.2)			3 (3.2)	3 (3.2)		
**Education, n (%)**			0.670†	0.043			0.512†	0.129			0.921†	0.017			0.771†	0.106
School leaver	197 (22.9)	182 (21.2)			44 (27.2)	37 (22.8)			198 (17.0)	204 (17.5)			17 (18.3)	14 (15.1)		
Further education	384 (44.7)	389 (45.2)			78 (48.1)	77 (47.5)			457 (39.1)	449 (38.4)			41 (44.1)	40 (43.0)		
Higher education	279 (32.4)	289 (33.6)			40 (24.7)	48 (29.6)			513 (43.9)	515 (44.1)			35 (37.6)	39 (41.9)		
**Deprivation Index, n (%)**			0.539†	0.085			0.394†	0.226			0.704†	0.061			0.065†	0.447
1	192 (22.3)	201 (23.4)			37 (22.8)	39 (24.1)			324 (27.7)	310 (26.5)			24 (25.8)	28 (30.1)		
2	199 (23.1)	197 (22.9)			30 (18.5)	41 (25.3)			280 (24.0)	274 (23.5)			18 (19.4)	18 (19.4)		
3	189 (22.0)	171 (19.9)			36 (22.2)	26 (16.0)			252 (21.6)	242 (20.7)			23 (24.7)	12 (12.9)		
4	149 (17.3)	171 (19.9)			37 (22.8)	31 (19.1)			186 (15.9)	210 (18.0)			11 (11.8)	23 (24.7)		
5	131 (15.2)	120 (14.0)			22 (13.6)	25 (15.4)			126 (10.8)	132 (11.3)			17 (18.3)	12 (12.9)		
**Wear season, n (%)**			0.530†	0.072			0.909†	0.082			0.722†	0.048			0.463†	0.237
Fall	270 (31.4)	264 (30.7)			47 (29.0)	51 (31.5)			314 (26.9)	324 (27.7)			21 (22.6)	29 (31.2)		
Winter	203 (23.6)	187 (21.7)			31 (19.1)	27 (16.7)			227 (19.4)	243 (20.8)			28 (30.1)	21 (22.6)		
Spring	183 (21.3)	207 (24.1)			31 (19.1)	29 (17.9)			295 (25.3)	278 (23.8)			15 (16.1)	17 (18.3)		
Summer	204 (23.7)	202 (23.5)			53 (32.7)	55 (34.0)			332 (28.4)	323 (27.7)			29 (31.2)	26 (28.0)		

Standardized mean differences (SMDs) are shown for assessment of post-matching balance.

* Age at the time when the participant wore the accelerometer wearable.

† Pearson’s chi-squared test.

‡ Kruskal-Wallis rank sum test.

d/w, days per week; HR, hip resurfacing; OA, osteoarthritis; THA, total hip arthroplasty; TKA, total knee arthroplasty; UKA, unicompartmental knee arthroplasty.

**Table II.** Participant characteristics before and after propensity score matching in knee and hip arthroplasty cohorts. Baseline characteristics of participants undergoing knee (TKA vs UKA) and hip (THA vs HR) arthroplasty before and after 4:1 propensity score matching.

Characteristic	Knee cohort				Hip cohort			
	TKA (n = 648)	UKA (n = 162)	p-value	SMD	THA (n = 372)	HR (n = 93)	p-value	SMD
**Sex, n (%)**			0.916†	0.009			0.470†	0.084
Female	345 (53.2)	87 (53.7)			139 (37.4)	31 (33.3)		
Male	303 (46.8)	75 (46.3)			233 (62.6)	62 (66.7)		
Median age, yrs (IQR)*	68.0 (64.0 to 71.0)	67.0 (62.0 to 71.0)	0.227‡	0.185	65 (61 to 69)	64 (59 to 67)	0.160‡	0.147
**Median BMI, kg/m^2^ (IQR)**	29.4 (26.6 to 32.8)	28.7 (26.4 to 32.9)	0.402‡	0.056	27.3 (24.2 to 29.9)	27.3 (24.7 to 29.8)	0.949‡	0.017
**Ethnicity, n (%)**			0.597†	0.133			0.529†	0.099
White	637 (98.3)	158 (97.5)			367 (98.7)	91 (97.8)		
Asian or Asian British	6 (0.9)	3 (1.9)			0 (0)	0 (0)		
Black or Black British	1 (0.2)	0 (0.0)			4 (1.1)	1 (1.1)		
Mixed	2 (0.3)	1 (0.6)			1 (1.1)	1 (1.1)		
Other ethnic group	2 (0.3)	0 (0.0)			0 (0)	0 (0)		
**Median CCI score (IQR)**	0.00 (0.00 to 0.00)	0.00 (0.00 to 1.00)	0.388‡	0.015	0.00 (0.00 to 0.00)	0.00 (0.00 to 0.00)	0.509‡	0.071
**Alcohol consumption, n (%)**			0.874†	0.046			0.782†	0.091
Never	27 (4.2)	6 (3.7)			11 (3.0)	2 (2.2)		
< 3 d/w	279 (43.1)	67 (41.4)			128 (34.4)	29 (31.2)		
≥ 3 d/w	342 (52.8)	89 (54.9)			233 (62.6)	62 (66.7)		
**Smoking, n (%)**			0.106†	0.188			0.280†	0.195
Never	312 (48.1)	63 (38.9)			203 (54.6)	58 (62.4)		
Previous	297 (45.8)	87 (53.7)			145 (39.0)	32 (34.4)		
Current	39 (6.0)	12 (7.4)			24 (6.5)	3 (3.2)		
**Education, n (%)**			0.437†	0.114			0.940†	0.041
School leaver	131 (20.4)	37 (22.8)			153 (41.1)	40 (43.0)		
Further education	290 (44.8)	77 (47.5)			163 (43.8)	39 (41.9)		
Higher education	226 (34.9)	48 (29.6)			56 (15.1)	14 (15.1)		
**Deprivation Index, n (%)**			0.856†	0.102			0.228†	0.01
1	159 (24.5)	39 (24.1)			96 (25.8)	28 (30.1)		
2	145 (22.4)	41 (25.3)			79 (21.2)	18 (19.4)		
3	123 (19.0)	26 (16.0)			84 (22.6)	12 (12.9)		
4	130 (20.1)	31 (19.1)			68 (18.3)	23 (24.7)		
5	91 (14.0)	25 (15.4)			45 (12.1)	12 (12.9)		
**Wear season, n (%)**			0.495†	0.135			0.940†	0.073
Fall	210 (32.4)	51 (31.5)			105 (28.2)	29 (31.2)		
Winter	121 (18.7)	27 (16.7)			73 (19.6)	17 (18.3)		
Spring	135 (20.8)	29 (17.9)			100 (26.9)	26 (28.0)		
Summer	182 (28.1)	55 (34.0)			94 (25.3)	21 (22.6)		
**Median time from operation to accelerometer, mths (IQR)**	31 (13 to 58)	35 (14 to 60)	0.542‡	0.017	75 (35 to 121)	91 (67 to 122)	0.070‡	0.234

Standardized mean differences (SMDs) are shown for the assessment of post matching balance.

* Age at the time when the participant wore the accelerometer wearable.

† Pearson’s chi-squared test.

‡ Kruskal-Wallis rank sum test.

HR, hip resurfacing; OA, osteoarthritis; THA, total hip arthroplasty; TKA, total knee arthroplasty; UKA, unicompartmental knee arthroplasty.

### PA levels in knee operations compared to healthy controls

After 1:1 matching, TKA patients walked significantly fewer steps per day than controls (7,620 vs 8,281 steps/day, p < 0.001) and engaged in significantly less MVPA (0.30 vs 0.40 hrs/day, p < 0.001), with peak cadence also lower in TKA patients (107 vs 111 steps/min, p < 0.001). In contrast, UKA patients showed no significant differences from their matched controls across any PA metric, including steps/day (9,150 vs 8,937, p = 0.631), MVPA (0.41 vs 0.47 hrs/day, p = 0.639), peak cadence (p = 0.089), or ENMO (p = 0.722) (Table III).

**Table III.** Device-measured physical activity (PA) in knee arthroplasty cohorts compared to matched non-osteoarthritic (non-OA) controls. Values are reported as median (IQR).

Variable	1:1 PSM TKA vs Non OA			1:1 PSM UKA vs Non OA			4:1 PSM TKA vs UKA		
	TKA (n = 860)	Non OA (n = 860)	p-value (TKA vs Non OA)	UKA (n = 162)	Non OA (n = 162)	p-value (UKA vs Non OA)	TKA (n = 648)	UKA (n = 162)	p-value (TKA vs UKA)
Light PA (hrs/day)	4.73 (3.62 to 5.94)	4.57 (3.62 to 5.75)	0.113	4.90 (3.92 to 5.86)	4.86 (3.74 to 5.93)	0.469	4.83 (3.70 to 6.13)	4.90 (3.92 to 5.86)	0.636
Moderate-vigorous PA (hrs/day)	0.30 (0.12 to 0.62)	0.40 (0.18 to 0.77)	< 0.001	0.41 (0.17 to 0.84)	0.47 (0.21 to 0.77)	0.639	0.35 (0.14 to 0.65)	0.41 (0.17 to 0.84)	0.037
Avg adjusted daily steps	7,620 (5,524 to10,147)	8,281 (6,116 to10,861)	< 0.001	9,150 (7,139 to 11,432)	8,937 (6,554 to 11,480)	0.631	7,920 (5,881 to 10,362)	9,150 (7,139 to 11,432)	< 0.001
Peak adjusted cadence	107 (97 to 114)	111 (101 to 118)	< 0.001	110 (102 to 117)	112 (104 to 119)	0.089	107 (99 to 114)	110 (102 to 117)	0.002
ENMO, mg	24 (19 to 29)	24 (19 to 28)	0.489	25 (20 to 31)	25 (21 to 30)	0.722	24 (20 to 29)	25 (20 to 31)	0.408

p-values reflect between-group differences assessed via Kruskal–Wallis rank sum test.

ENMO, Euclidean Norm Minus One; PSM, propensity score matching; TKA, total knee arthroplasty; UKA, unicompartmental knee arthroplasty.

### Cross-sectional comparison of PA by time since surgery in knee patients

When stratified by time since surgery, UKA patients showed higher activity than TKA patients across both postoperative time periods (Table IV). In the first postoperative year, UKA patients walked 3,474 more steps/day than TKA patients (10,899 vs 7,425, p = 0.005) and had substantially higher MVPA (0.73 vs 0.29 hrs/day, p = 0.030) and ENMO (28 vs 24 mg, p = 0.010). At 12 months or beyond, UKA patients continued to walk more steps per day than TKA patients (9,021 vs 8,103, p = 0.008) and had higher peak cadence (111 vs 108 steps/min, p = 0.009), though differences in MVPA (p = 0.250) and ENMO (p = 0.708) did not reach statistical significance.

**Table IV.** Device-measured physical activity (PA) after unicompartmental knee arthroplasty (UKA) and total knee arthroplasty (TKA) stratified by time since surgery. Values are reported as median (IQR).

Variable	TKA < 12 mths (n = 145)	UKA < 12 mths (n = 31)	p-value*	TKA ≥ 12 mths (n = 530)	UKA ≥ 12 mths (n = 131)	p-value*
Light PA, hrs/day	4.58 (3.52 to 5.84)	5.14 (3.79 to 6.00)	0.248	4.86 (3.80 to 6.14)	4.79 (3.92 to 5.83)	0.882
Moderate-vigorous PA, hrs/day	0.29 (0.12 to 0.64)	0.73 (0.16 to 0.94)	0.030	0.38 (0.15 to 0.66)	0.40 (0.17 to 0.81)	0.250
Average adjusted daily steps	7,425 (5,288 to 9,773)	10,899 (5,677 to 12,758)	0.005	8,103 (6,071 to 10,588)	9,021 (7,228 to 11,081)	0.008
Peak adjusted cadence	104 (95 to 113)	107 (99 to 118)	0.087	108 (99 to 114)	111 (103 to 117)	0.009
ENMO, mg	24 (20 to 28)	28 (24 to 31)	0.010	24 (21 to 30)	25 (20 to 30)	0.708

* Kruskal-Wallis rank sum test.

ENMO, Euclidean Norm Minus One.

### Direct comparison of TKA and UKA

After 4:1 propensity score matching, UKA patients walked approximately 1,230 more steps/day than TKA patients (9,150 vs 7,920, p < 0.001) and had higher peak cadence (110 vs 107 steps/min, p = 0.002). UKA patients also engaged in more MVPA (0.41 vs 0.35 hrs/day, p = 0.037). Differences in light PA (p = 0.636) and ENMO (p = 0.408) did not reach statistical significance (Table III). To account for potential residual confounding between groups after matching, a linear regression model was fitted. The model confirmed that UKA patients walked an estimated 903 more steps/day than TKA patients (95% CI 343 to 1,463, p = 0.002) and demonstrated higher peak cadence (*β* = 2.80 steps/min, 95% CI 0.82 to 4.70, p = 0.005). The difference in MVPA did not reach statistical significance (*β* = 0.07 hrs/day, 95% CI −0.01 to 0.15, p = 0.099) (Table V).

**Table V.** Linear regression adjusted differences between total and partial arthroplasties of knees and hips.

Metric	Knees		Hips	
	Estimate (95% CI)	Adj. estimate (95% CI)	Estimate (95% CI)	Adj. estimate (95% CI)
Light PA, hrs/day	0.17 (-0.11 to 0.44) (p = 0.233)	0.00 (-0.26 to 0.26) (p = 0.997)	-0.19 (-0.53 to 0.15) (p = 0.275)	-0.09 (-0.43 to 0.26) (p = 0.615)
Moderate-vigorous PA, hrs/day	0.12 (0.03 to 0.21) (p = 0.007)	0.07 (-0.01 to 0.15) (p = 0.099)	0.29 (0.16 to 0.41) (p < 0.001)	0.12 (0.00 to 0.24) (p = 0.048)
Average adjusted daily steps	1,454 (847 to 2,061) (p < 0.001)	903 (343 to 1,463) (p = 0.002)	1,526 (751 to 2,302) (p < 0.001)	799 (34 to 1,564) (p = 0.041)
Peak adjusted cadence	4.20 (2.10 to 6.20) (p < 0.001)	2.80 (0.82 to 4.70) (p = 0.005)	3.20 (0.58 to 5.90) (p = 0.017)	2.20 (-0.47 to 4.90) (p = 0.106)
ENMO, mg	0.96 (-0.47 to 2.4) (p = 0.190)	-0.25 (-1.60 to 1.10) (p = 0.720)	2.30 (0.61 to 4.00) (p = 0.008)	1.00 (-0.73 to 2.70) (p = 0.258)

Adjusted linear models for sex, age, BMI, season of accelerometer wear, alcohol consumption, Charlson Comorbidity Index score, and time from operation to accelerometer worn.

ENMO, Euclidean Norm Minus One.

### PA levels in hip operations compared to healthy controls

After 1:1 PSM, HR patients showed no significant differences from their matched controls across any PA metric (Table VI). In contrast, THA patients walked significantly fewer steps per day than controls (8,750 vs 9,036, p < 0.001), engaged in less MVPA (0.44 vs 0.50 hrs/day, p = 0.002), and had lower peak cadence (111 vs 113 steps/min, p = 0.010). Corresponding visual comparisons are shown in Figure 2b.

**Table VI.** Device-measured physical activity (PA) in hip arthroplasty cohorts compared to matched non-osteoarthritic (non-OA) controls. Values are presented as median (IQR).

Variable	1:1 PSM THA vs Non OA			1:1 PSM HR vs Non OA			4:1 PSM THA vs HR		
	THA (n = 1,168)	Non OA (n = 1,168)	p-value THA vs Non OA*	HR (n = 93)	Non OA (n = 93)	p-value HR vs Non OA*	THA (n = 372)	HR (n = 93)	p-value THA vs HR*
Light PA, hrs/day	5.02 (4.01 to 6.08)	4.92 (3.88 to 5.94)	0.169	4.70 (3.89 to 5.90)	4.87 (3.41 to 5.97)	0.794	4.89 (3.66 to 5.97)	4.70 (3.89 to 5.90)	0.972
Moderate-vigorous PA, hrs/day	0.44 (0.18 to 0.79)	0.50 (0.24 to 0.90)	0.002	0.63 (0.35 to 0.97)	0.57 (0.29 to 0.91)	0.474	0.57 (0.25 to 1.01)	0.63 (0.35 to 0.97)	0.280
Average adjusted daily steps	8,750 (6,464 to 11,128)	9,036 (6,983 to 11,728)	< 0.001	10,112 (7,986 to 12,658)	10,246 (7,019 to 11,803)	0.321	9,139 (6,802 to 11,671)	10,112 (7,986 to 12,658)	0.032
Peak adjusted cadence	111 (102 to 119)	113 (104 to 120)	0.010	114 (109 to 119)	115 (106 to 121)	0.886	112 (104 to 119)	114 (109 to 119)	0.025
ENMO, mg	25 (21 to 30)	25 (21 to 31)	0.446	28 (23 to 33)	25 (21 to 31)	0.119	26 (21 to 31)	28 (23 to 33)	0.090

* Kruskal-Wallis rank sum test.

ENMO, Euclidean Norm Minus One; HR, hip resurfacing; PSM, propensity score matching; THA, total hip arthroplasty.

**Fig. 2:**
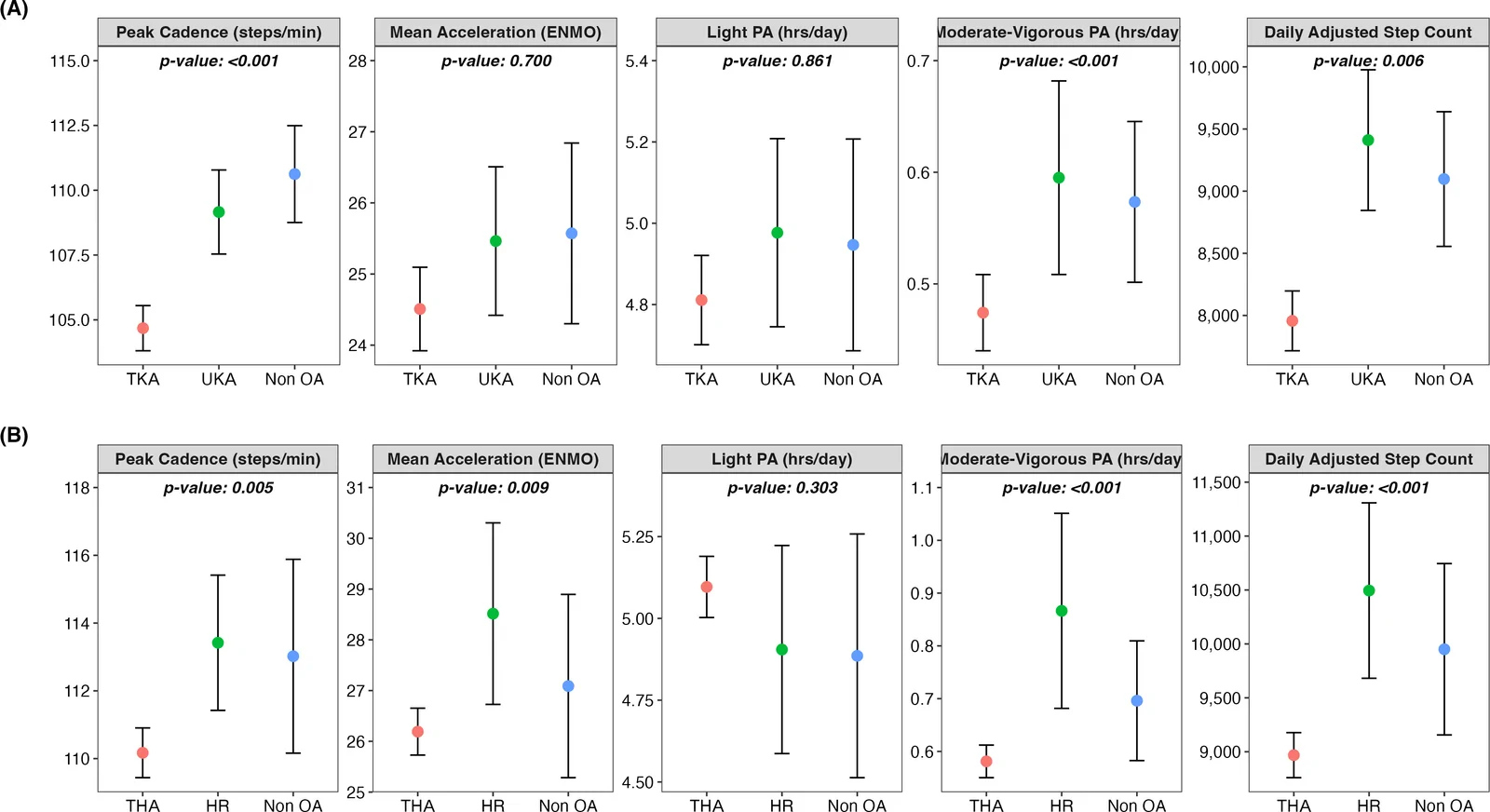
Device-measured physical activity (PA) across arthroplasty cohorts compared to non-osteoarthritic controls. a) Comparison of total knee arthroplasty (TKA), unicompartmental knee arthroplasty (UKA), and matched non-osteoarthritic (Non OA) controls. b) Comparison of total hip arthroplasty (THA), hip resurfacing (HR), and matched Non OA controls. The mean values of the measures alongside the 95% CIs are displayed. p-values were derived using the Kruskal-Wallis rank sum test to assess overall group differences within each metric. Outcomes include: peak cadence (steps/min), mean acceleration (Euclidean Norm Minus One (ENMO)), light physical activity (hrs/day), moderate-to-vigorous PA (MVPA, hrs/day), daily adjusted step count.

### Cross-sectional comparison of PA by time since surgery in hip patients

As all HR patients were at least 12 months post-surgery, early recovery comparisons were not feasible. When looking at the late recovery group (≥ 12 months), HR patients continued to outperform THA in PA (Table VII). HR patients walked a median of 10,112 steps/day compared to 9,129 steps/day in THA patients (p = 0.037). Peak cadence was also higher in HR patients (114 vs 112 steps/min, p = 0.027), while differences in MVPA (p = 0.348) and ENMO (p = 0.084) did not reach statistical significance. Temporal changes following hip arthroplasty are depicted in Figure 3c.

**Table VII.** Physical activity (PA) after hip resurfacing (HR) and total hip arthroplasty (THA) stratified by time since surgery. Values are reported as median (IQR).

Variable	THA ≥ 12 mths (n = 372)	HR ≥ 12 mths (n = 93)	p-value*
Light PA, hrs/day	4.89 (3.65 to 5.99)	4.70 (3.89 to 5.90)	0.987
Moderate-vigorous PA, hrs/day	0.57 (0.26 to 1.05)	0.63 (0.35 to 0.97)	0.348
Average adjusted daily steps	9,129 (6,837 to 11,718)	10,112 (7,986 to 12,658)	0.037
Peak adjusted cadence	112 (104 to 119)	114 (109 to 119)	0.027
ENMO, mg	26 (22 to 31)	28 (23 to 33)	0.084

* Kruskal-Wallis rank sum test.

ENMO, Euclidean Norm Minus One.

**Fig. 3:**
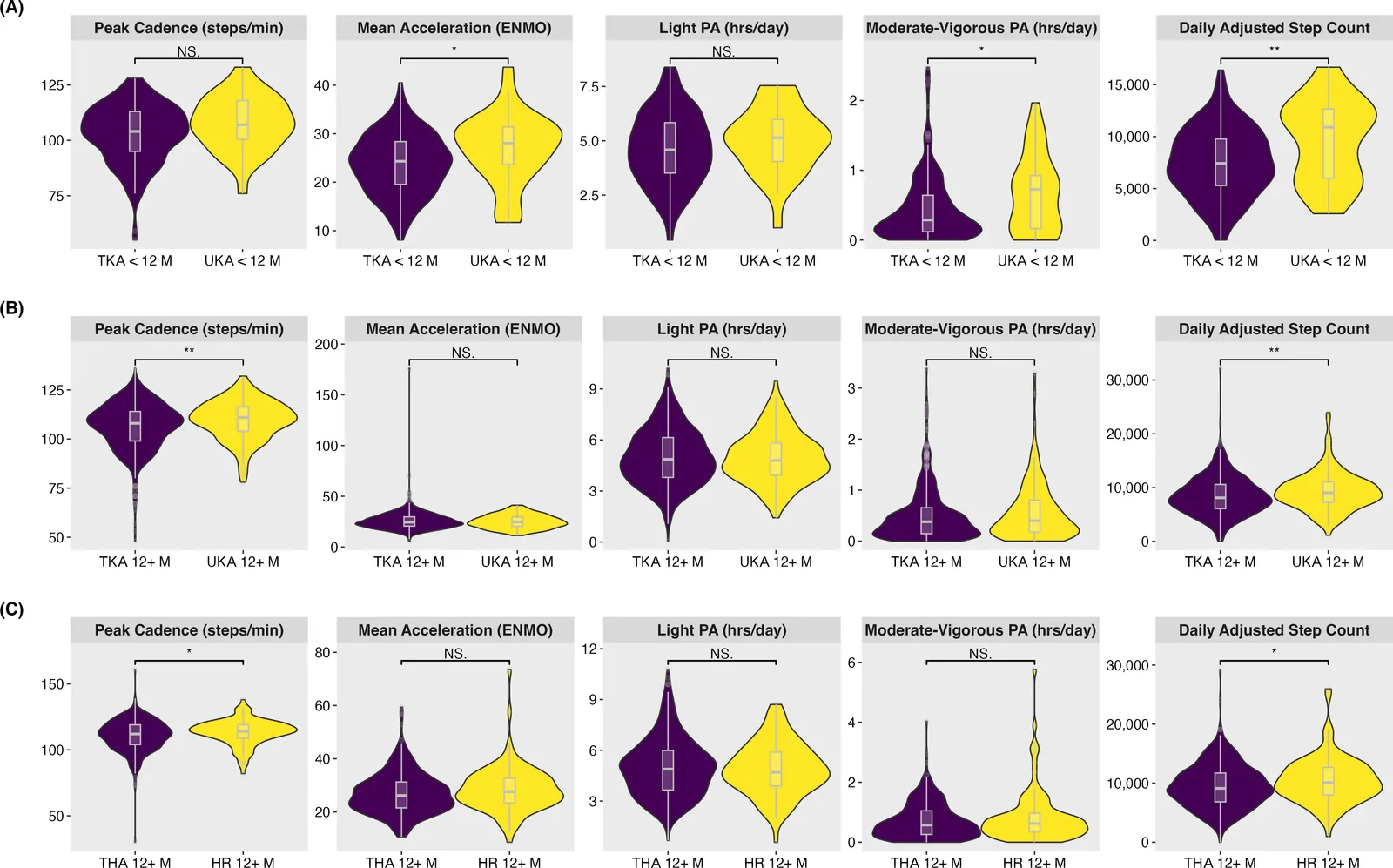
Device measured physical activity across arthroplasty cohorts stratified by postoperative time period. a) Comparison of total knee arthroplasty (TKA) and unicompartmental knee arthroplasty (UKA) within the first 12 postoperative months. b) Comparison of TKA and UKA after the first 12 postoperative months. c) Comparison of total hip arthroplasty (THA) and hip resurfacing (HR) after the first 12 postoperative months. Outcomes include: peak cadence (steps/min), mean acceleration (Euclidean Norm Minus One (ENMO)), light physical activity (PA) (hrs/day), moderate-to-vigorous PA (MVPA, hrs/day), daily adjusted step count. *p < 0.05. NS, non-significant.

### Direct comparison of THA vs HR before and after PSM

After 4:1 PSM, HR patients walked approximately 973 more steps/day than THA patients (10,112 vs 9,139, p = 0.032) and had higher peak cadence (114 vs 112 steps/min, p = 0.025). Differences in MVPA (p = 0.280), light PA (p = 0.972), and ENMO (p = 0.090) did not reach statistical significance (Table VI). After adjustment for covariates, HR patients walked an estimated 799 more steps/day (95% CI 34 to 1,564, p = 0.041), and showed higher adjusted MVPA (estimate: 0.12 hrs/day, 95% CI 0.00 to 0.24, p = 0.048). The adjusted difference in peak cadence did not reach statistical significance (p = 0.106) (Table V).

## Discussion

This study used accelerometer-derived data from the UK Biobank to examine postoperative physical activity following four major joint arthroplasty procedures. Consistent with prior work by Small et al,^15^ we found that individuals who had undergone arthroplasty were generally less active than non-arthritic controls. Building on this, our study identified that HR patients achieved step counts and cadence comparable to matched healthy controls, while THA patients demonstrated significantly lower activity. In the knee cohort, UKA patients showed activity levels closer to controls, whereas TKA patients had consistently lower step counts, cadence, and MVPA. All surgical groups fell within the ‘somewhat active’ to ‘active’ category (7,500 to 12,499 steps/day), as defined by Tudor-Locke and Bassett,^31^ with HR patients achieving a median of 10,112 steps/day, surpassing the 10,000-step threshold generally associated with an ‘active’ lifestyle.^31-33^ When comparing procedures directly, HR and UKA demonstrated superior ambulatory outcomes compared to their conventional counterparts (THA and TKA), respectively.

Our findings demonstrate that UKA is associated with superior postoperative PA compared to TKA, reinforcing the hypothesis that joint-preserving interventions offer functional advantages by better replicating native knee kinematics. While both groups met the PA threshold recommended by the Physical Activity Guidelines for Americans (≥ 150 minutes of moderate-intensity activity per week), UKA patients consistently exhibited higher daily step counts and cadence.^34^

After matching, UKA patients walked approximately 1,230 more steps/day than TKA patients and demonstrated higher peak cadence (110 vs 107 steps/min, p = 0.002). To account for residual confounding after matching, a linear regression model was fitted, which confirmed that UKA patients walked an estimated 903 more steps/day (95% CI 343 to 1,463, p = 0.002) and had higher peak cadence (*β* = 2.80 steps/min, 95% CI 0.82 to 4.70, p = 0.005), while the difference in MVPA did not reach statistical significance after adjustment (*β* = 0.07 hrs/day, p = 0.099). These findings are consistent with temporospatial gait analyses from Çankaya et al,^35^ who reported a five-step/min advantage for UKA over TKA.^35^ Median MVPA was 172 minutes/week in the UKA group versus 147 minutes in TKA, exceeding guideline thresholds but highlighting a functional gap. Prior work using ActivPAL sensors (Axivity AX3 triaxial accelerometer, Axivity, UK) has reported that TKA often failed to meet these activity targets.^36^ The discrepancy may reflect differences in device design; ActivPAL, which focuses on posture and step counting, may underestimate intensity or walking time in post-surgical patients.^37^ The clinical relevance of these differences requires careful interpretation. While the between-group cadence differences were statistically significant, their absolute magnitude was modest and may not represent a perceptible functional difference for every patient. By contrast, the approximately 900 additional steps/day observed after regression adjustment may be more clinically meaningful, given prior literature linking higher habitual step volume to improved cardiovascular and mortality outcomes.^38,39^

From a clinical perspective, these findings suggest that UKA recipients may achieve activity levels closer to those of non-arthritic controls when selected appropriately. Prior evidence from PROMs has consistently demonstrated greater satisfaction and function with UKA at one year and beyond.^40^ Our study extends this by demonstrating corresponding objective differences in real-world PA. Nevertheless, improved short- to medium-term mobility must be weighed against long-term implant longevity. While registry data suggest that UKA survivorship may exceed 96.5% at ten years in younger populations,^41^ pooled data continue to report lower overall survival relative to TKA (90% vs 97.5% at five years, and 70% vs 89% at 15 years).^42^ This trade-off between function and risk of revision should be considered in the patient selection, particularly in younger, active individuals who may prioritize short- to medium-term performance but who also face a longer cumulative risk of revision.

After matching, HR patients walked a median of 973 more steps/day than THA patients and demonstrated higher peak cadence (114 vs 112 steps/min, p = 0.025). To account for residual confounding after matching, a linear regression model was fitted, which confirmed that HR patients walked an estimated 799 more steps/day (95% CI 34 to 1,564, p = 0.041) and had higher MVPA (*β* = 0.12 hrs/day, 95% CI 0.00 to 0.24, p = 0.048), while the adjusted difference in peak cadence did not reach statistical significance (p = 0.106). This regression-confirmed difference in activity remains clinically relevant, surpassing thresholds commonly associated with meaningful health benefits including reduced cardiovascular risk and lower all-cause mortality.^38,39^

This clinically relevant and statistically significant difference in activity may provide an explanation for the reduced mortality following these less invasive procedures reported from registry data.^5,43^ Nonetheless, our study was not designed to assess mortality and cannot determine whether PA explains previously reported survival differences. Exercise has recently been firmly linked to reduction in all-cause mortality, death from cardiovascular causes, and death from malignant disease.^44^ The size of the reduction seen in this meta-analysis of over 30 million patients is similar to that seen in these patient groups, providing a good explanation for the differences in mortality reported by both the NJR and the AOANJRR.^45^

Prior studies have suggested that superior activity levels and mortality observed following HR may be attributed to residual confounding.^46^ In our study, HR demonstrated superior ambulatory outcomes even after PSM.^47,48^ This is consistent with prior randomized evidence showing more favourable gait characteristics at higher walking speeds after HR, although our observational findings cannot isolate procedure effect from residual confounding.^6^ Biomechanically, HR preserves the femoral neck and uses a larger femoral head, which better replicates natural hip anatomy. This allows for more physiological load transfer across the joint and preserves proprioception, contributing to a more stable, controlled gait. The use of a larger femoral head also provides inherent enhanced joint stability, and reduced risk of dislocation.^49^ Beyond mechanical stability, systemic factors may well contribute. A bidirectional Mendelian randomization study by Zhu et al^50^ identified 11 causal associations between circulating biomarkers and OA, finding that certain metabolic markers like low-density lipoprotein-cholesterol and glucose are causally linked to OA risk. By preserving the native femoral neck and more of the joint’s original architecture, bone-preserving procedures minimize the systemic inflammatory disruptions that often accompany the more invasive total arthroplasties, thereby facilitating a smoother return to high-level activity.^50^

These findings suggest that HR recipients may tolerate higher-demand ambulatory activity, although the extent to which this reflects procedure mechanics versus preoperative patient characteristics cannot be determined from this study.

A key strength of this study is the use of device-measured PA in a large population-based cohort. PSM was employed to address baseline characteristics differences between groups and to enable clinically interpretable comparisons. Furthermore, although MVPA is often estimated using self-reported measures in population-based research, MVPA in this study was derived objectively from accelerometer data within the UK Biobank, thereby reducing recall and reporting bias.^51,52^ However, several limitations must be acknowledged. First, PA was measured over a single seven-day period, which may not reflect habitual behaviour and is susceptible to short-term fluctuations such as illness or adverse weather. Nonetheless, a seven-day accelerometer protocol is widely used in large epidemiological cohorts and methodological studies, which indicate that approximately five to seven monitoring days are sufficient to obtain reliable estimates of weekly habitual PA and to capture both weekdays and weekend days.^53-55^ Second, the cross-sectional design precludes assessment of within-subject change from pre- to post-surgery, limiting our ability to draw conclusions about recovery trajectories. Third, surgical factors such as implant type, surgical approach, experience of operating surgeon, and fixation method, were not available, which may influence generalizability to patients undergoing more contemporary procedures. This is particularly relevant for UKA and HR, where advances in implant design may offer improved outcomes. Additionally, adherence to postoperative rehabilitation protocols was not assessed. Variability in physiotherapy attendance, engagement, and compliance may have influenced activity outcomes and introduced further unmeasured confounding, which can only be properly addressed in prospective or randomized trial incorporating wearable monitoring. Finally, patients selected for UKA and HR are often younger, fitter, and more likely to have higher preoperative activity potential than those selected for TKA or THA. Moreover, procedure choice may reflect surgeon-level decision-making factors, including training, experience, preference, and local thresholds for offering bone-preserving procedures, rather than objective disease severity alone.^56^ Although PSM reduces imbalance in measured covariates, it cannot fully eliminate residual confounding by indication or surgeon selection bias. As such, the observed associations may reflect both procedure-related factors and underlying differences in patient selection. Despite these limitations, the use of objective accelerometer-derived outcomes, consistency across multiple activity metrics, and concordance between matched and regression-based analyses support the robustness of the observed associations.

In conclusion, this study set out to determine how PA differs following total and bone-preserving hip and knee arthroplasty, using objective accelerometry data. Compared to their total joint counterparts, patients undergoing UKA and HR exhibited higher daily step counts and cadence, closely approximating levels observed in healthy controls. In contrast, TKA and THA patients showed persistent deficits in PA.

By directly comparing surgical subtypes using propensity-matched analyses, this study provides evidence that bone-preserving procedures are associated with higher real-world PA levels than total arthroplasty in selected patients. These differences underscore the importance of considering long-term activity potential, not just implant survival, when selecting arthroplasty procedures for active individuals. In doing so, the study addresses the central question posed and reinforces the broader conclusion that procedure type may contribute to differences in PA after joint arthroplasty.

## Data Availability

The raw datasets analysed in the current study are not publicly available from the authors, as access is governed by UK Biobank data access requirements. Access to the underlying data is restricted to approved researchers who have obtained the necessary permissions from UK Biobank. Derived analyses and aggregate results may be made available by the corresponding author upon reasonable request, subject to applicable UK Biobank data governance requirements. Further inquiries can be made to the corresponding author.
